# Group I introns: Structure, splicing and their applications in medical mycology

**DOI:** 10.1590/1678-4685-GMB-2023-0228

**Published:** 2024-03-25

**Authors:** Ronald Muryellison Oliveira da Silva Gomes, Kássia Jéssica Galdino da Silva, Raquel Cordeiro Theodoro

**Affiliations:** 1 Universidade Federal do Rio Grande do Norte, Instituto de Medicina Tropical do Rio Grande do Norte, Natal, RN, Brazil.; 2 Universidade Federal do Rio Grande do Norte, Centro de Biociências, Departamento de Biologia Celular de Genética, Natal, RN, Brazil.

**Keywords:** Autocatalytic introns, RNA, mitochondrial genome, pathogenic fungi

## Abstract

Group I introns are small RNAs (250-500 nt) capable of catalyzing their own splicing from the precursor RNA. They are widely distributed across the tree of life and have intricate relationships with their host genomes. In this work, we review its basic structure, self-splicing and its mechanisms of gene mobility. As they are widely found in unicellular eukaryotes, especially fungi, we gathered information regarding their possible impact on the physiology of fungal cells and the possible application of these introns in medical mycology.

## Introduction

**Figure 1 -:**
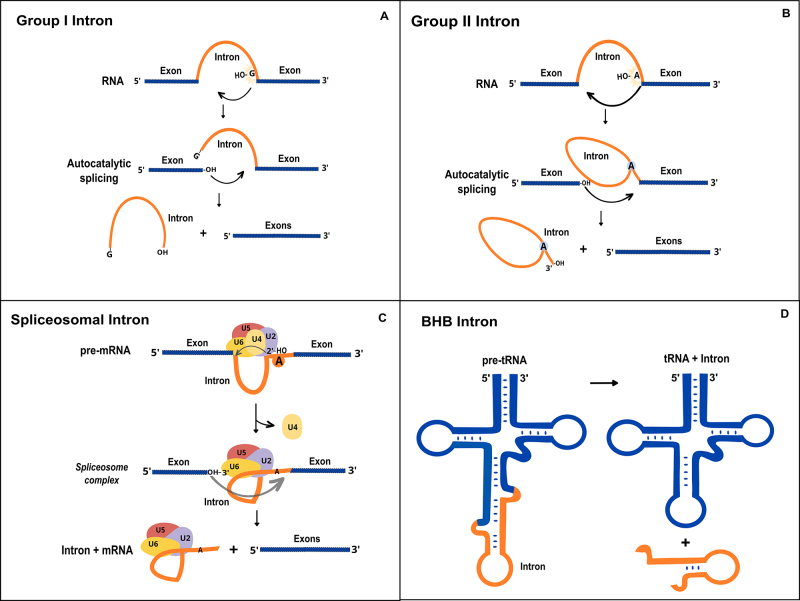
The four different classes of introns. (a) group I introns. (b) group II introns. (c) Spliceosomal introns. (d) BHB introns. Only group I and II introns are autocatalytic. Adapted from Mukhopadhyay and Hausner (2021), Lai et al. (2021), Randau and Söll (2008).

Initially considered “junk” DNA, introns are currently defined as non-coding sequences within genes and are viewed as important genetic elements, since they increase transcriptome and proteome diversity, perform regulatory activities in the cell, affect gene expression, RNA processing, degradation, and messenger RNA translation (Ignatenko et al., 2019). Based on tertiary structure and mechanism of their excision, introns are classified into four main categories: (i) spliceosomal, (ii) group I self-splicing, (iii) group II self-splicing, and (iv) tRNA introns or archaeal introns (also known as BHB introns) (Hausner et al., 2014) (Figure 1). 

Group I introns, the focus of this review, are small RNAs ranging in size from 250 to 500 nucleotides (Haugen et al., 2005). These introns are categorized as primary examples of catalytic RNAs and are formally recognized as group I ribozymes, due to their ability to execute RNA cleavage and ligation as part of their splicing process. Some of them exhibit a highly complex organization, presenting functional genes and other sequences, establishing deep relationships with the genomes of their hosts (Nielsen and Johansen, 2009). In addition to their self-splicing ability, group I introns can encode specific DNA homing endonucleases (HEs) that make them mobile elements within genomes through double-strand DNA breaks (DSB) and homologous recombination (Megarioti and Kouvelis, 2020).

Despite being known to the scientific community for more than 40 years (Cech et al., 1981), group I introns have been poorly studied and were considered mere genetic parasites for a long time. This simplistic view existed because it was known that (i) theoretically, these small RNA molecules were capable of self-splicing from the RNA without the assistance of any protein, thus not influencing the expression of their host gene, and (ii) some of these genetic elements could be mobile, capable of being “copied” to a homologous or cognate allele lacking the intron, leading to a super-Mendelian inheritance characterized by an increased frequency of alleles containing the intron in the population. In other words, these introns increase in frequency in the population not through genetic drift or natural selection, but through a genomic mobility mechanism.

With the advances in molecular biology and the efforts towards sequencing the nuclear and mitochondrial genomes of various species, it has become possible to acquire more knowledge about these introns, such as their distribution in genes and genomes, their secondary structure, and their classification into different families. Additionally, possible functions of these elements have been explored, considering their coevolution with host genomes, which would directly affect the modulation of gene expression in their host organisms.

In view of the wide distribution of group I introns in the Kingdom Fungi, the aim of this work was to review the basic mechanisms of splicing and mobility of group I introns and how such mechanisms could impact cell physiology, specially virulence, in pathogenic fungi.

## Group I introns: Where they occur and how they splice out 

In the early 1980s, it was discovered that an intronic structure in a precursor rRNA of *Tetrahymena thermophila* could undergo splicing without the assistance of proteins (Cech et al., 1981). For the first time scientists recognized that RNAs could possess catalytic activities derived from their specific secondary structures, thus playing more diverse roles in biological processes than previously thought. A new field of study emerged, focusing on what was called autocatalytic group I introns.

**Figure 2 -:**
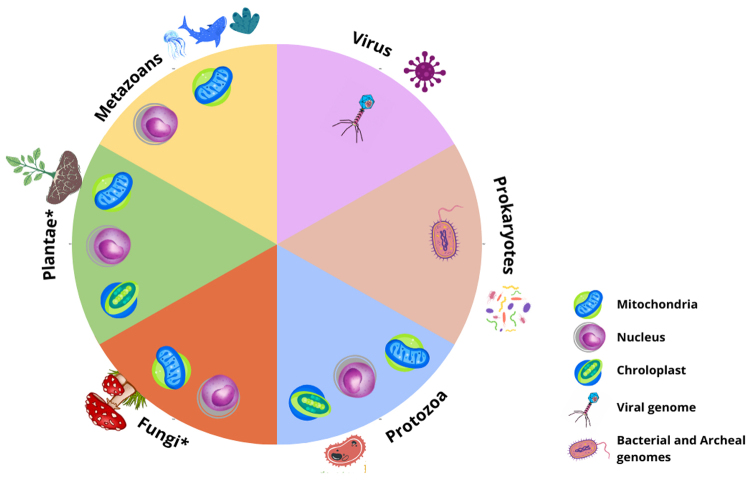
Occurrence of group I introns in different genomes and cellular compartments. The asterisks emphasize lineages within the tree of life that are notably rich in group I introns, encompassing fungi, plants, and red and green algae. In metazoans, group I introns have been found in non-bilaterian basal lineages (sponges, cnidarians, and placozoans) and in some species of sharks (Corsaro and Venditti, 2020; Mukhopadhyay and Hausner, 2021).

Group I Introns occur in all life domains. In bacteria, they are primarily found in rRNA and tRNA genes but are less common in coding genes. In some protists, plants, and fungi, these introns can be found in nuclear rRNA genes as well as in rRNA, tRNA, and protein-coding genes of organelles, such as fungal mitochondria and plant mitochondria and chloroplasts (Rogers, 2019; Hausner et al., 2014; Haugen et al., 2005). The branches of the tree of life known to date as exhibiting a notable abundance of group I introns include fungi, plants, as well as red and green algae. These specific groups account for approximately 90% of all the identified group I Introns (Haugen *et al*., 2005; Hausner *et al*., 2014). They are absent in metazoans, except for a few non-bilateral basal lineages and five shark species (Figure 2) (Corsaro and Venditti, 2020; Mukhopadhyay and Hausner, 2021).

**Figure 3 -:**
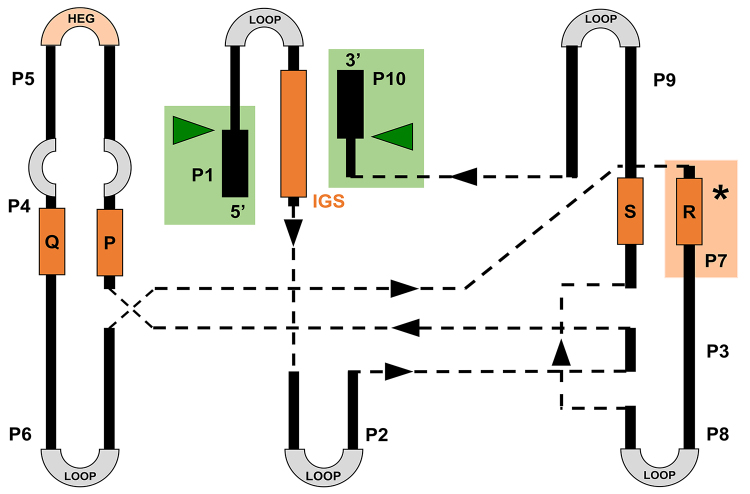
Generic representation of the group I intron structure with emphasis on key loops and domains featuring conserved sequences. Gray regions indicate unpaired segments (loops) that may contain Open Reading Frames (ORFs), which eventually harbor homing endonuclease genes (HEGs, shown in red). Orange rectangles show the location of conserved sequences P, Q, R, S, and IGS. Black rectangles represent exons located in the upstream and downstream regions of the 5’ and 3’ splice sites (green triangles), respectively. The binding site for exogenous guanine (ExoG) on the G-binding site, located in loop P7, is represented by an asterisk (adapted from Hausner et al., 2014).

Although the group I introns are not highly conserved in their primary sequence, crystallographic studies of three group I introns have revealed that the three-dimensional structures of their catalytic cores are remarkably similar (Vicens and Cech, 2006). The catalytic core is located at the junction of the P4-P6 (P4, P5, P6) and P3-P9 (P3, P7, P8, P9) domains, formed by intramolecular base pairing (Figure 3). P1 and P10 define the 5’ and 3’ splicing sites, respectively, which are formed by base pairing between an internal guide sequence (IGS) located immediately upstream of the 5’ splicing site and an exon sequence adjacent to the splicing site. The final nucleotide of the intron, called ωG, and the first nucleotide of the 3’ exon define the 3’ splicing site, and the interaction between the adjacent portion of the IGS and the first nucleotide of the 3’ exon constitutes P10 (Adams et al., 2004). Other elements around the conserved catalytic core are important for ribozyme stability and proper folding (Engelhardt et al., 2000).

Based on conserved core domains, variation in the configuration of secondary structure elements, the presence of peripheral elements and characteristics of the P7:P7’ helix, group I introns have been classified into five main groups (IA, IB, IC, ID, IE), which were further subdivided according to specific structural features, resulting in 17 subgroups: IA1, IA2, IA3, IB1, IB2, IB3, IB4, IC1, IC2, IC3, ID1, ID2, ID3, IE1, IE2, IE3, IE4 (Cinget and Bélanger, 2020).

**Figure 4 -:**
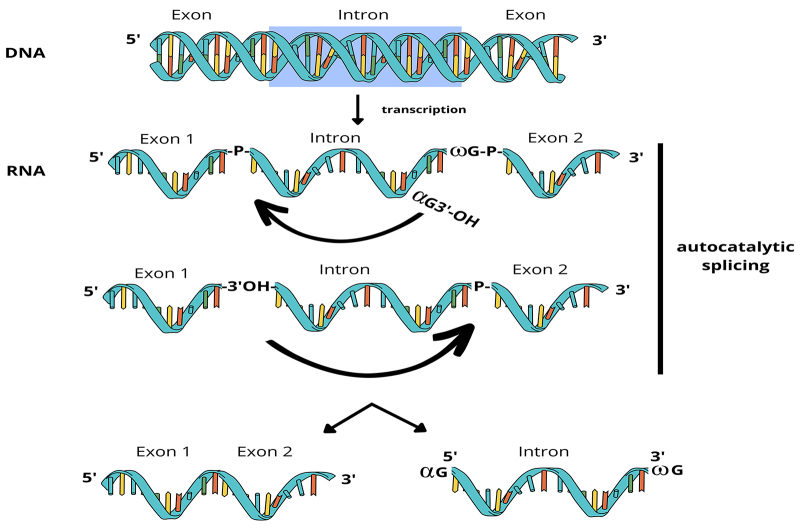
Schematic representation of group I intron splicing. The splicing pathway involves two consecutive transesterification reactions. The first reaction is initiated by the 3’-OH group of an exogenous GTP (αG) that binds to the ωG-binding pocket located in the P7 region, and the 3’-OH group attacks the 5’ splice site. In the second reaction, the 3’-OH group of the released 5’ exon attacks the phosphodiester bond between the intron terminal G (ωG) and the 3’ exon, resulting in the release of the intron and the joining of the exons (adapted from Haugen et al., 2005).

These introns self-splice through two consecutive transesterification reactions, initiated when the substrate P1 helix correctly folds into the catalytic core of the ribozyme in response to the binding of a exogenous guanosine (ExoG) to the folded catalytic core, resulting in intron excision and joining of the flanking exons (Figure 4) (Haugen et al., 2005).

Many studies have shown that, in some cases, the efficiency of *in vivo* splicing of these elements requires the action of proteins with maturase functions, which can be encoded by the intron itself, as in the case of some homing endonucleases that have acquired this function (Belfort, 2003), or by other proteins encoded by the host organism genome. For instance, certain nuclear encoded proteins with RNA-binding domains appear to have acquired an additional function, which is to assist in the correct folding and splicing of these introns. Examples of such proteins include RNase II, tRNA synthetase, and RNA helicase (Akins and Lambowitz, 1987; Mohr et al., 2002; Jarmoskaite and Russell, 2011).

## Mobility mechanisms of group I introns

**Figure 5 -:**
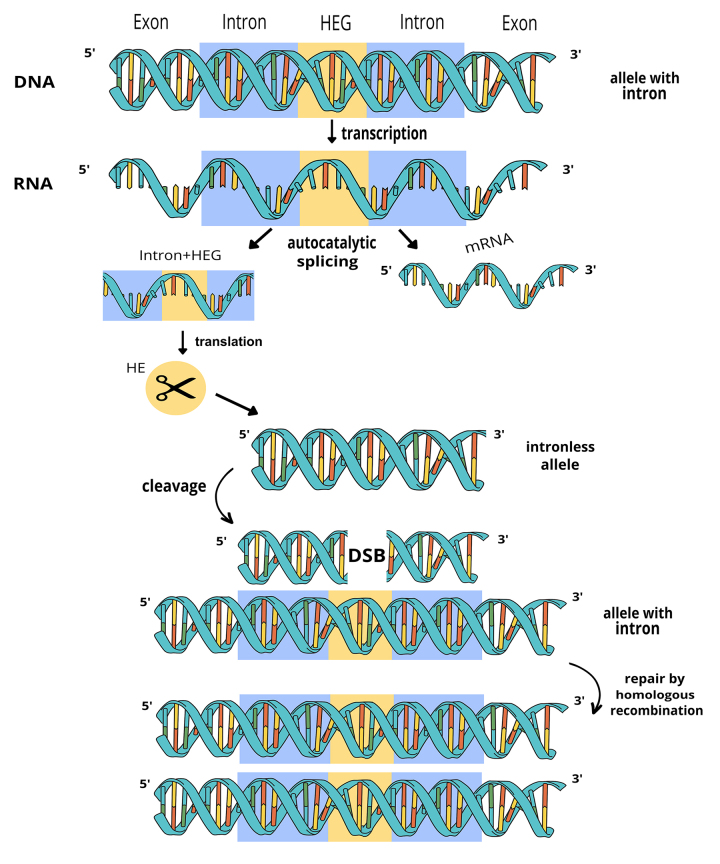
‘Homing’ process of a group I intron. In this DNA-based mobility pathway, the allele with IGI (shaded in blue) expresses an endonuclease (in beige). Cleavage at the recognition site of the endonuclease in the homologous allele without the intron causes DNA damage. The double-strand break (DSB) activates the cellular mechanism of homologous recombination repair, copying the intron and the endonuclease gene to the new site. Adapted from Barzel et al. (2011).

Some group I introns also encode homing endonucleases (HE), which promote a double-strand break in a cognate empty allele, copying themselves into homologous sites through homologous recombination. Thus, through a gene conversion mechanism (rather than natural selection), alleles containing the intron increase in frequency in the population (Figure 5). Once most loci are occupied, the functionality of the HE becomes a neutral trait since there are no more sites to invade, as the presence of the intron disrupts the recognition site of the HE. Consequently, degenerate homing endonucleases may increase in frequency through simple genetic drift, potentially leading to the loss of the intron in the population. This phenomenon allows empty alleles to re-emerge and be invaded, completing what is called “the homing cycle” (Goddard and Burt, 1999). Alternatively, some homing endonucleases escape the degeneration stage by assuming new and important roles, such as maturases that assist in the splicing of their own host intron (Belfort, 2003). Their maintenance in the population is then ensured by natural selection, as correct splicing is necessary for the host RNA to function and, therefore, vital for the host organism.

It is estimated that approximately one-fourth or one-third of group I introns and a similar fraction of group II introns contain internal ORFs, and a significant number of these ORFs appear to be mobile (Chevalier and Stoddard, 2001). In eukaryotes, homing endonucleases (HEs) are found in nuclear, mitochondrial, and chloroplast genomes. They are classified into four families based on conserved amino acid motifs that participate in the enzyme’s active site: GIY-YIG (GIY), LAGLIDADG (LD), His-Cys box, and HNH (Stoddard, 2014). However, in fungal mtDNA, only genes that encode GIY and LD endonucleases can be found (Belfort et al., 2002; Megarioti and Kouvelis, 2020). Differences in their sequences suggest independent origins for all of them (Taylor and Stoddard, 2012).

It is evident that introns are not universally distributed, as closely related sister species can differ in the presence of these elements, while distantly related species can share the same intron. Furthermore, some of these elements can occupy heterologous sites (non-cognate alleles). This sporadic distribution indicates successive losses of these elements in different lineages, as well as their horizontal transfer (Zhang *et al*., 2015).

Furthermore, it is observed that the same group I intron can invade different genes or even different regions of the same gene. This observation is likely not due to the action of homing endonucleases, as they are site-specific. However, another mechanism associated with the invasion of group I introns into different sequences of the genome, known as reverse splicing, seems to be responsible for their intra- and intergenic mobility. In reverse splicing, the free spliced group I intron inserts itself into another RNAs through base complementarity of 4-6 nucleotides between the intron and the RNA exon (Hedberg and Johansen, 2013), much shorter and therefore less specific than the site recognized by the HE. Then, the recombined RNA is reverse transcribed to a complementary DNA (cDNA) by any reverse transcriptase in the cell (from group II introns, transposons or retrovirus). This cDNA sequence can be copied into cognate allele by homologous recombination (Birgisdottir and Johansen, 2005). This mechanism has been proposed to explain the phylogenetic proximity of group I introns originating from heterologous sites, and the strongest evidence occurs in rRNA gene introns (Bhattacharya et al., 2005).

Still, we cannot rule out the nonspecificity of some HEs as a cause of ectopic integration. The “promiscuity” (low specificity) of HE has been experimentally reported, for example, for the VDE endonuclease of *Saccharomyces cariocanus* (Posey et al., 2004). It is now known that the specificity of HE can be influenced by certain stressful conditions, such as the presence of reactive oxygen species (Robbins et al., 2011). It has also been proposed that mutations in HEGs lead to new recognition sequences, promoting intron transposition to heterologous sites (Mullineux et al., 2011). For instance, it has been proven that substitutions with the amino acids E or D into the two metal-binding residues of the LAGLIDADG homing endonucleases make the cleavage profile broader than the wild-type meganucleases (McMurrough et al., 2018). Equally important for the targetability of such homing endonucleases are the substitutions in the recognition sequence. Lambert et al. (2016) showed that the cleavage capacity of LAGLIDADG is limited by highly specific indirect recognition sites.

Although reverse splicing facilitates intron mobility between distinct genes, which would be more difficult to occur through the homing process, the latter is still more efficient in promoting intron mobility, as it does not depend on additional steps required by reverse splicing, such as reverse transcription (Bhattacharya et al., 2005).

## Distribution of group I introns in fungal mitogenomes and their potential biological significance

Group I introns are believed to have an irregular and highly selective distribution in nature, both in terms of the microbial species where they are found and the genes they invade. According to data from Comparative RNA Web (CRW) (https://crw2-comparative-rna-web.org/crw1_legacy/), until the year 2014, a number of 2,939 group I introns had been reported, with 2,851 found in eukaryotes. Among these, 2,223 are in the nucleus and 628 in organelle genomes (393 in chloroplasts and 235 in mitochondria). In fungi, CRW reported a total of 1,656 group I introns, with 1,508 in the nucleus and 148 in the mitochondria. So far, described nuclear group I introns are restricted to rRNA genes (mostly LSU and SSU, with few occurrences in the ITS regions), while in organelles, they can be found in regions encoding rRNA, tRNA, and proteins (Corsaro and Venditti, 2020).

Megarioti and Kouvelis (2020) pointed out that mobile introns are frequently found in conserved mitochondrial genes, such as those related to ATP production (ATP synthase subunits - *atp6, atp8*, and *atp9*), oxidative phosphorylation (NADH dehydrogenase subunits - *nad1-6* and *nad4L*), apocytochrome b *(cob*), cytochrome C oxidase (*cox1-3*), and rRNA (*rnl* and *rns*). The study identified 487 introns (IGI and IGII) encoding endonucleases and highlighted that the genes richest in introns are cox1 (50% of the total), cob (17%), nad5 (7.5%), and rnl (6.5%).

The more pronounced presence of these introns in the *cox1*, *cob* and *nad5* genes is, at the very least, intriguing, as these genes play important roles in the electron transport chain (ETC). The *cox1* gene encodes subunit 1 of cytochrome c oxidase (Complex IV), which participates in the final stage of the respiratory chain, responsible for transferring electrons from cytochrome c to molecular oxygen, thereby reducing it to H_2_O, while *cob* gene encodes cytochrome b, an essential component of the functional unit of Complex III (ubiquinol-cytochrome c oxidoreductase) in the ETC. The *nad5* gene encodes the core subunit of NADH dehydrogenase, Complex I, which functions in the transfer of electrons from NADH to the respiratory chain. Proper functioning of these genes is crucial for cellular respiration (Fox, 2012).

Regarding the *rnl* gene, which is transcribed into the large subunit ribosomal RNA, its essentiality for mitochondrial function is indisputable, as it forms part of the structure of mitoribosomes. Rogers (2019) proposed that ribosomal RNAs and group I introns have a long shared evolutionary history. The author reviewed and analyzed rRNA intron sequences, their locations, structural characteristics, and splicing mechanisms, suggesting that rRNA gene loci may have served as evolutionary nurseries for the formation and diversification of introns.

Instead of being primarily disruptive elements (as commonly perceived), autocatalytic introns may have played significant roles since the early stages of rRNA and ribosome evolution, sequentially assembling functional RNAs during the evolutionary process of rRNA formation. Rogers (2019) highlights that group I and II introns are found in the oldest parts of rRNAs, suggesting primitive origins, while other classes of introns are found only in parts of the rRNA that evolved later. Characterizations of rRNAs in the three major domains of life indicate that introns and rRNAs have long and integrated evolutionary histories, which have had important influences on genome, organism, and organelle evolution (Rogers, 2019). After endosymbiosis, in the mitochondrial environment, nonspecific (heterologous) invasions from existing introns in the rRNA genes of the ancestral prokaryote may have contributed to the rapid colonization of almost all mitochondrial genes.

## Impact of the presence of group I introns in fungal genomes

The influence exerted by group I introns on their host can be initially observed in how they determine the size of the mitochondrial genome. Within the Fungi Kingdom, it is well-documented that mitochondrial DNA (mtDNA) exhibits a wide range of sizes mainly because of the presence or absence of self-catalytic introns, spanning from 11kb in *Hanseniaspora uvarum* to over 200 kb in *Morchella importuna* (Pramateftaki et al., 2006; Liu et al., 2020). Nevertheless, the understanding of the mechanisms driving intron acquisition and loss remains limited. It is also unclear why, in certain cases, organellar genomes are entirely devoid of introns, or in extreme scenarios, introns constitute a substantial portion of the genome’s nucleotide content (Mukhopadhyay and Hausner, 2021).

An investigation into mitogenome polymorphisms in the pathogenic fungus *Cryptococcus neoformans* revealed that mtDNA size in this species complex can vary from 24,740 to 31,327 base pairs (bp). This variability is primarily attributed to differences in the number and size of mitochondrial introns, which can result in up to a 20% disparity in mitogenome size among cryptic species of *C. neoformans* (Wang and Xu, 2020). 

In 2018, Rudan *et al*. conducted an experiment evaluating the mitochondrial morphophysiology of two strains of *Saccharomyces cerevisiae*. One strain lacked introns in its mitogenome, while the other strain contained the 13 possible mitochondrial introns existing in this species (1 IGI in the *rnl* gene, 7 in *cox1*, and 5 in *cob*). The authors observed that the absence of mitochondrial introns led to a tubular morphology due to increased transcription of nuclear genes encoding mitofusin (*fzo1*) and GTPase (*mgm1*), which are the main regulators of mitochondrial fusion, encoded in the nucleus. Furthermore, the absence of these introns resulted in higher mitochondrial activity with increased oxygen consumption and cellular ATP levels during exponential growth. However, mitochondrial superoxide levels were reduced due to high expression of *sod2*. Thus, intron removal challenges, but does not compromise, mitochondrial function. The transcription of *cox1* and *cob* in the intron-less strain was significantly elevated, triggering a retrograde response in mitochondria. This signaling pathway involves various factors that detect and transmit mitochondrial signals to effect changes in nuclear gene expression, leading to metabolic reconfiguration to compensate for mitochondrial dysfunction (Jazwinski, 2013). Therefore, in *S. cerevisiae*, the inefficient intron processing has become an integral part of normal mitochondrial gene expression. This study was of significant importance as it demonstrated that the presence of introns directly influences gene expression, particularly for *cox1* and *cob* genes.

Group I introns play a significant role in generating diversity in mtDNA (Hamari et al., 2002). Studies have shown that mobile introns have the most significant impact on the interaction between mitochondrial genomes, with their homing process generating recombinant mtDNAs. Mitochondrial DNA rearrangements caused by intron movements were previously reported by Earl et al. (1981) when mitochondrial recombinations between closely related *Aspergillus* species were detected. The interspecific recombinants differed from the parental strains only in the presence or absence of introns primarily located in the *cox1* gene.

In another study that utilized genomic data from populations of *S. cerevisiae*, *Schizosaccharomyces pombe*, and *Lachancea kluyveri*, non-random patterns of genetic diversity were found in exons flanking autocatalytic introns (Repar and Warnecke, 2017). It was observed that, for all three species, the density of polymorphisms in exons is higher at sites near mobile introns, suggesting that the mobility of these genetic elements is a direct driver of host gene diversity, possibly through two mechanisms: (i) endonuclease activity and subsequent error-prone repair leave a mutational “scar” in intron flanking sequences, or (ii) non-random patterns of genetic diversity are caused by exonic conversion during horizontal gene transfer events (e.g., hybridization between two species, one with an intron and another without).

Obviously, the mobility of these elements, which is directly responsible for their increased frequency in the population, encounters its limit in the fitness of the host organism. The mutational burden in the vicinity of the intron insertion site would affect cellular fitness. Thus, the mutagenic effect of intronic mobility restricts the number of possible insertion sites to evolutionarily sustainable locations (Repar and Warnecke, 2017).

The mitogenome can significantly affect the virulence potential of fungi. Human fungal pathogens, such as those in the *Aspergillus*, *Candida*, and *Cryptococcus* genera, are obligate aerobes. Therefore, mitochondrial morpho-physiology plays an essential role in their survival, both in the environment and during infection, when oxygen concentrations can vary in different host tissues (Ma et al., 2009). Rudan et al. (2018) studied the influence of autocatalytic introns on the expression of mitochondrial genes, which in turn modulates their physiology and morphology. These aspects are certainly related to the virulence of animal pathogenic fungi; however, there is no evidence that group I introns impact directly on the virulence of these pathogens. On the other hand, for the phytopathogenic fungus *Cryphonectria parasitica*, the presence of a splicing-defective group II intron proved to be directly responsible for the hypovirulence of some strains (Baidyaroy et al., 2011). 

## Variability of Group I Introns in pathogenic fungal species and its possible application for differentiation of fungal species or genotypes

Considering the difficulty in treating fungal diseases, especially those of a systemic nature, the search for new therapeutic targets is urgent. Current targets often lead to severe side effects and commonly require patient hospitalization. Additionally, the correct identification of fungal species and genotyping of isolates have become increasingly important, given that there are differences in susceptibility to antifungals among different genotypes.

Based on the above, the search for new molecular markers for genotyping, fungal virulence, and drug susceptibility is necessary. Self-splicing group I introns seem to fulfill the requirements for this task because (i) they are polymorphic in their presence and base pair sequence, (ii) their self-splicing is known to be inhibited by some drugs, (iii) their correct splicing under parasitic conditions is indispensable for the pathogen’s survival and (iv) they are not present in mammals.

Regarding the presence of group I introns in pathogenic fungi, in *C. albicans*, the intron located in the 25S rRNA gene is used for genotyping, through a PCR-RFLP system (Karahan et al., 2012). In the *Histoplasma* genus, an autocatalytic intron has been characterized in the nuclear 18S gene (Lasker et al., 1998). However, no population study has been conducted to address whether this intron can differentiate cryptic species within the *Histoplasma capsulatum* complex. 

In pathogenic *Cryptococcus*, the distribution and polymorphism of group I introns were described in the mitochondrial genes *mtLSU* or 23S (Gomes et al., 2018) and *cob* and *cox1* (Gomes *et al*., 2023) of approximately 80 isolates belonging to different genotypes. These studies showed that *C. neoformans* species complex has fewer introns compared to *C. gattii* complex and differentiating the cryptic species using a single intron is not feasible. Nevertheless, it was possible to distinguish genotypes within each species complex by combining PCRs of *mtLSU* and *cox1* introns for *C. neoformans* species, and *mtLSU* and cob introns for *C. gattii* species (Gomes *et al*., 2023). 

## Group I introns as therapeutic targets against fungi 

As well reviewed by Vicens and Westhof (2022), Childs-Disney et al. (2022) and Johnson (2010), RNA plays a vital role in cellular processes and the growing understanding of the structural and functional diversity of RNAs reveals that many of them have regulatory activities related to their structure, and their dysfunction can lead to diseases. Additionally, the drugability of RNA, like proteins, allows for the development of drugs targeting specific sites on RNA. Two approaches to target RNA involve inhibiting ribozyme catalysis or using ribozymes to correct defective RNA, acting as gene therapy or gene inhibitors. This strategy is effective, especially considering ribozymes are present in pathogens but not in humans. 

In recent years, there has been a convergence of methods for screening, optimizing drugs, and validating targets, resulting in the approval of several molecules that target RNA for clinical applications. However, selecting the appropriate target for developing RNA-targeted drugs poses challenges. Not all RNA can be readily targeted due to the dynamic nature of many RNA structures. The validation of suitable RNA targets is a time-consuming process, akin to the procedures for identifying protein targets. The efficacy of RNA targeting is heightened when there is a comprehensive understanding of the diverse functional structures that RNA can assume. Thus, RNAs with known natural ligands become preferred targets, as endogenous ligands act by altering local molecular transitions or changes between conformations, providing a basis for drug discovery. Most current efforts in RNA drug targeting, particularly in the context of antimicrobials, concentrate on developing compounds that bind to well-characterized sites in riboswitches and ribosomal RNA (Blount and Breaker, 2006; Wilson, 2014).

Concerning the splicing process of group I introns, it is known that it faces inhibition through distinct mechanisms, as elucidated by Wallis and Schroeder (1997): some compounds engage in a competition with the guanosine cofactor for the guanosine binding site, such as antibiotics like streptomycin (von Ahsen and Schroeder, 1990, 1991), certain tuberactinomycins (Wank et al., 1994), specific pseudodisaccharides (Rogers and Davies, 1994) and L-arginine (Liu and Leibowitz, 1995). On the other hand, non-competitive inhibition has been documented for aminoglycosides such as neomycin B, tobramycin, gentamicin, and 5-epi-sisomycin (von Ahsen *et al*., 1991, 1992), along with pentamidine and tetracycline (Liu *et al*., 1994). Although a detailed exploration of how these antibiotics obstruct splicing is pending, various observations suggest their interference with catalytic metal ions (Wallis and Schroeder, 1997). 

The auto-splicing of group I introns in fungi is an important drug target since inhibiting splicing prevents the precursor RNA from becoming functional. As the host RNAs containing these introns are essential for basic cell metabolism, the failure to excise the intron would have a significant impact on the viability of the fungal cell. Importantly, this is a safe target as these invasive elements are absent in the human genome (Malbert et al., 2023; Disney et al., 2001). This is particularly relevant due to the increase in reported cases of fungal diseases, especially among opportunistic pathogens that affect immunocompromised patients, as well as the rise in antifungal resistance, which make the search for new drugs and therapeutic targets urgent (Fisher et al., 2022).

The presence of auto-splicing group I introns is linked to susceptibility to pentamidine and 5-flucytosine. For instance, several studies have shown the effect of pentamidine on autocatalytic introns *in vitro* for *Pneumocystis carinii* (Liu et al., 1994), *Candida albicans* (Miletti and Leibowitz, 2000; Zhang et al., 2002), and *Saccharomyces cerevisiae* (Zhang *et al*., 2000). The inhibitory potential of pentamidine on the splicing of the 25S rRNA intron of *C. albicans* (designated Ca.LSU) has been demonstrated both in vitro and in fungal culture. Furthermore, the cellular splicing inhibition was found to be correlated with the differential growth inhibition of a strain with group I introns compared to a closely related strain without these introns, suggesting that the inhibition of intron autocatalysis is a possible mechanism of action of this medication (Miletti and Leibowitz, 2000). Pentamidine also demonstrated efficacy in inhibiting the *in vitro* splicing of group I introns from *Coxiella burnetii*, a pathogenic bacterium that causes Q fever in humans (Minnick *et al*., 2010).

Subsequent studies that incubated the Ca.LSU intron with pentamidine *in vitro* suggested that it directly binds to group I introns and may induce an altered P7 helix structure (which contains the active site). It was also found that positively charged molecules (magnesium, calcium, spermidine) antagonize the catalysis inhibition by the drug and alleviate its effect on ribozyme folding, suggesting that pentamidine binds to one or more magnesium binding site(s) within the intron to exert its inhibitory effect (Zhang et al., 2002).

In *Candida albicans*, it has been shown that 5-flucytosine, which is a fluorinated pyrimidine analog (5-fluorocytosine), inhibits auto-splicing of the nuclear 25S gene’s group I intron due to the insertion of fluorouracil into its precursor RNA (Król et al., 2019; Mercure et al., 1993). In *in vitro* susceptibility tests with 40 strains, Gomes et al. (2018) reported that isolates of *C. neoformans* and *C. gattii* lacking introns in the mitochondrial mt*LSU* gene had, on average, higher MIC values for 5-flucytosine (*p*=0.059), reinforcing the idea that there is a relationship between the presence of group I introns and increased sensitivity to this drug in *Cryptococcus* as well.

Modified small oligonucleotides have also demonstrated inhibitory action on auto-splicing through mechanisms of suicidal inhibition, where the oligonucleotide binds to the precursor RNA and subsequently inhibits the intron’s auto-splicing *in vivo* (Zhang et al., 2009; Disney et al., 2004). Similarly, several aminoglycosides and peptidic antibiotics have been reported as inhibitors of group I intron auto-splicing (von Ahsen et al., 1992). Bleomycin, an antitumor glycopeptide antibiotic, has been shown to interact with the group I intron in *C. albicans*, affecting its conformation and inhibiting its auto-splicing (Jayaguru and Raghunathan, 2007). A strong correlation between the presence of the intron and high susceptibility to bleomycin is evident, as the minimum inhibitory concentration of bleomycin for strains containing the intron was 1.56 μg/mL, whereas for those lacking the intron, it was 6.25 μg/mL. 

**Figure 6 -:**
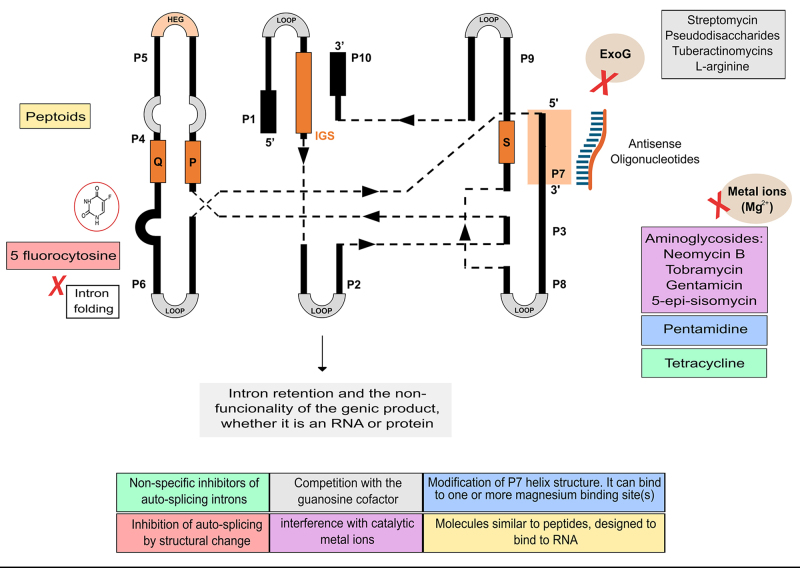
Molecules that inhibit group I intron splicing *in vitro* and their potential mechanisms of action. Streptomycin (von Ahsen and Schroeder, 1990, 1991), specific tuberactinomycins (Wank et al., 1994), certain pseudodisaccharides (Rogers and Davies, 1994), and L-arginine (Liu and Leibowitz, 1995) competitively engage with the guanosine cofactor, while aminoglycosides such as neomycin B, tobramycin, gentamicin, 5-epi-sisomycin (von Ahsen *et al*., 1991, 1992), along with pentamidine and tetracycline (Liu *et al*., 1994), exhibit non-competitive inhibition but with a function not yet well elucidated (aminoglycosides likely interfere with the binding to ionic cofactors). Furthermore, modified small oligonucleotides inhibit auto-splicing by binding to precursor RNA specifically at the P7 site (Zhang et al., 2009; Disney et al., 2004). The 5-flucytosine is a base analog that, when incorporated into the intron, disrupts its folding (Mercure et al., 1993) and peptoids are synthetic oligomers, similar to peptides, designed to bind RNAs and disrupt their folding (Labuda et al., 2009). .

Peptoids are also among the molecules that can be used to inhibit the splicing of group I introns. These compounds, synthetic oligomers similar to peptides, have side chains attached to the nitrogen atoms of the backbone, providing structural and functional diversity. In a study conducted by Labuda et al. (2009) using microarrays, peptoids that inhibit the group I intron RNA from *C. albicans* were identified. The most potent inhibitor from this study, composed of phenylguanidine and tryptamine submonomers, was six times more effective than pentamidine. These findings underscore the ability to identify modulators of RNA function by utilizing RNA-focused chemical libraries, employing statistical analysis to identify binding features, and incorporating these features into second-generation inhibitors to enhance ligand potencies. Figure 6 illustrates some molecules known to inhibit the splicing of group I introns, along with their presumed mechanisms of action.

Figure 6 - 

Recently, Malbert et al. (2023) developed a trans-splicing high-throughput screening strategy to search for safe and efficient new antifungal compounds. They found that group I introns, such as the group ID intron found in *Fusarium oxysporum*, a phytopathogenic fungi, and an opportunistic animal pathogen (Nag *et al*., 2022), can be adapted in a high-throughput screening to find new antifungal compounds. In this study, the authors designed a construct with this intron to function as a trans-acting ribozyme and employed a fluorescence-based reporter system to monitor its real-time splicing activity, paving the way for studying the drugability of these introns in pathogenic fungi affecting plants and animals. This advancement enhances the discovery of small molecules that selectively bind to group I introns in future high-throughput screenings.

## Perspectives of studies on fungal group I introns

Advances in understanding mitochondrial function relevant to medical mycology occur primarily in four directions: (i) the role of mitochondrial morphology in virulence, (ii) mitochondrial genetics, with a focus on mitogenome recombination and mitochondrial inheritance, (iii) the role of mitochondria in drug resistance, and (iv) the interaction of mitochondria with other organelles and the nucleus (Verma et al., 2018). Only in recent years the relationship between the presence of introns and mitochondrial function has begun to be studied, but much remains to be elucidated in this research field.

The theme of autocatalytic genetic elements, especially group I introns, is of utmost importance in the field of medical mycology. These elements are absent from our genome, and their self-splicing is vital for fungal cells, making them attractive therapeutic targets. Additionally, the highly polymorphic nature of these elements, due to their evolutionary dynamics, makes them potential molecular markers useful for species-specific or genotype-specific diagnosis of certain pathogenic fungi.

Thus, these elements can open up new treatment strategies through splicing inhibition, improve diagnostics, aid in inferring antifungal resistance, and help understand differences in virulence, ultimately enhancing the prognosis of various fungal diseases, especially systemic mycoses, which are often overlooked. Additionally, understanding the natural conditions that may affect their self-splicing will certainly expand our knowledge of gene expression regulation in unicellular eukaryotes, such as fungi.
